# Low autocrine interferon beta production as a gene therapy approach for AIDS: Infusion of interferon beta-engineered lymphocytes in macaques chronically infected with SIVmac251

**DOI:** 10.1186/1742-4690-1-29

**Published:** 2004-09-25

**Authors:** Wilfried Gay, Evelyne Lauret, Bertrand Boson, Jérome Larghero, Franck Matheux, Sophie Peyramaure, Véronique Rousseau, Dominique Dormont, Edward De Maeyer, Roger Le Grand

**Affiliations:** 1 CEA, Laboratoire d'Immuno-Pathologie Expérimentale, Service de Neurovirologie, CRSSA, EPHE, IPSC, Université Paris XI, 18 route du Panorama 92265 Fontenay aux Roses, Cedex, France; 2 INSERM U362, Institut Gustave Roussy, 39 rue Camille Desmoulins, 94805 Villejuif, France; 3 Institut Fédératif de Neurobiologie Alfred Fessard CNRS UPR 9040 91198 Gif-sur-Yvette cedex, France

## Abstract

**Background:**

The aim of this study was to evaluate gene therapy for AIDS based on the transduction of circulating lymphocytes with a retroviral vector giving low levels of constitutive macaque interferon β production in macaques chronically infected with a pathogenic isolate of SIVmac251.

**Results:**

Two groups of three animals infected for more than one year with a pathogenic primary isolate of SIVmac251 were included in this study. The macaques received three infusions of their own lymphocytes transduced ex vivo with the construct encoding macaque IFN-β (MaIFN-β or with a vector carrying a version of the MaIFN-β gene with a deletion preventing translation of the mRNA. Cellular or plasma viremia increased transiently following injection in most cases, regardless of the retroviral construct used. Transduced cells were detected only transiently after each infusion, among the peripheral blood mononuclear cells of all the animals, with copy numbers of 10 to 1000 per 10^6 ^peripheral mononuclear cells.

**Conclusion:**

Long-term follow-up indicated that the transitory presence of such a small number of cells producing such small amounts of MaIFN-β did not prevent animals from the progressive decrease in CD4^+ ^cell count typical of infection with simian immunodeficiency virus. These results reveal potential pitfalls for future developments of gene therapy strategies of HIV infection.

## Background

Highly active antiretroviral therapy (HAART) effectively inhibits human immunodeficiency virus (HIV) replication, but it has been suggested that a combination of HAART and strategies for boosting the immune system would give more effective long-term control of HIV infection [1,2]. Interferon β (IFN-β) is an attractive candidate for such therapy: 1) it is a natural, potent antiviral protein that inhibits HIV at various stages of the viral cycle, from uptake to the release of virus particles [3-9]; 2) Type I IFNs display immunomodulatory properties that could improve the immune control of HIV replication [10-12].

During HIV infection, the induction of type I IFN production has been shown to be impaired in T cells and macrophages, which are considered to be the major targets of the virus [13-16]. However, the use of recombinant IFN in therapeutic strategies is limited by its poor bioavailability and the need for high doses to obtain an antiviral effect, resulting in deleterious side effects [17].

It has been suggested that the efficacy of type I IFNs for the treatment of HIV infection could be increased by developing a gene therapy strategy based on the modified production of IFN-β in genetically engineered lymphocytes [18]. For this purpose, a retroviral vector derived from Moloney murine leukemia virus, in which the human IFN-β coding sequence has been placed under the control of a fragment of the murine H2-K^b ^gene promoter, has been used to ensure the continuous generation of low levels of IFN-β in transduced cells [10,19]. The transduction of peripheral blood lymphocytes (PBL) with this vector inhibits HIV replication in vitro and increases the survival of CD4^+ ^cells in culture. Furthermore, IFN-β production in PBL from HIV-infected donors increases Th1-type cytokine production, improves cytotoxic responses against cells expressing HIV proteins, and the proliferative response to recall antigens [10,12]. These in vitro results have been confirmed in the SCID mouse model of HIV infection [20]. However, as the human-SCID mouse has a number of limitations as a model of AIDS, the efficacy and safety of this strategy should also be evaluated in a more appropriate model, such as macaques infected with simian immunodeficiency virus (SIV).

SIV resembles HIV-1 and HIV-2 in its genomic organization and biological properties [21] and systematically causes a disease in macaques that is remarkably similar to AIDS in humans [22]. We have previously shown that PBL obtained from seronegative animals and transduced with a vector carrying the macaque IFN-β coding sequence placed under the control of a 0.6-kb fragment of the murine H2-K^b ^gene promoter develop greater resistance to SIVmac251 in vitro [23]. In healthy seronegative macaques, infusion with autologous lymphocytes transduced ex vivo with the vector encoding IFN-β results in approximately 1 transduced cell per thousand peripheral blood mononuclear cells (PBMCs). The genetically modified cells were detected for at least 74 days after infusion, with no major side effects, in these experiments. Following infection with SIVmac251, macaques that had received the IFN-β construct infusion displayed lower peak plasma viral loads during primary infection than did control macaques. No adverse reaction was observed, and these macaques maintained high CD4+ T-lymphocyte counts for at least 478 days [24].

However, a gene therapy strategy for HIV infection would only be possible during the chronic phase of infection. At this stage, the immune system, and particularly CD4^+ ^T cells – the major target of our gene therapy approach – may be strongly affected by the virus. We therefore investigated the safety and efficacy of this strategy in macaques chronically infected with a primary, pathogenic isolate of SIVmac251, but still in an asymptomatic state. The efficacy of our strategy has been examined according to two parameters. The eventual survival advantage of IFN-β transduced cells has been monitored by following the presence of such transduced cells in the blood stream as well as in the lymph nodes of infused macaques. This group of animals was compared to a controlgroup having received cells transduced with a retrovirus carrying a modified version of the MaIFN sequence with a deletion blocking mRNA translation. Animals were subjected to three infusions of autologous T lymphocytes transduced ex vivo with both constructs. The eventual clinical benefits of the presence of IFN-β-transduced cells have been monitored for two years, by examining, in both groups of animals, the absolute number of circulating CD4+ lymphocytes, cell associated viral load and plasma vial load.

## Results

### Status of animals before treatment

The in vivo safety and anti-SIV efficacy of IFN-β-engineered lymphocytes in chronically SIV-infected macaques was assessed by following for two years animals that had received three infusions day 0, day 361 and day 613) of autologous T lymphocytes transduced with a construct encoding IFN-β (macaques IFN1, IFN2, IFN3) or, as a control, with a retrovirus carrying a modified version of the IFN-β that could not generate functional protein (macaques C1, C2, C3). These macaques had been infected with 4 AID_50 _of a primary, pathogenic isolate of SIVmac251 more than one year before the start of the experiment. On day 0, the mean number of circulating CD4^+ ^T lymphocytes was 767 ± 215 μl, and all animals had detectable SIV provirus in PBMCs (Table 1). Plasma SIV viremia was low or undetectable in most animals, the detection threshold being 1,500 copies of SIV RNA copies per milliliter of plasma.

**Table 1:** Immunological and virological parameters of macaques at day 0 of the experiment. At the onset of the experiment, the six male cynomolgus macaques (Macaca fascicularis) were chronically infected by 4 AID _50 _of a primary and pathogenic isolate of SIVmac251 for more than one year. They have been characterized for their mean number of circulating CD4+ T-lymphocytes, the time after SIV inoculation, and the cellular and plasma SIV viral loads. IFN group is represented by the three macaques that received their own cells transduced by the biologically active construct of IFN-β gene whereas the control group is represented by the three macaques that received their own cells transduced by the control construct. a: Immunophenotyping of Ficoll-purified PBMCs was performed by immunostaining with specific anti-CD4 and anti-CD8 antibodies, and analyzing by flow cytometry. The mean number of circulating CD4+ T-lymphocytes was determined at day 0 post first infusion with five points preceeding the onset of the experiment. b: Cellular viral load was estimated by a quantitative limit dilution nested PCR method allowing specific double amplification of a gagfragment of SIVmac251. Number of proviral copies was estimated by the last dilution that can display, in an agarose gel, a signal amplification. The number of SIVmac251 gag gene copies per 1 mg of DNA, for instance 131300 cells, was then brought back to a number of gene copies per 10^6 ^cells. c Plasma SIV viral load was determined by the branched-DNA method.

		Mean number of circulating CD4^+ ^lymphocytes ^a^	Time after inoculation of SIVmac251	Mean number of SIV proviral DNA copies in PBMCs ^b ^(Copies per 10^6 ^cells)	Plasma SIV load ^c^(10^3 ^copies per ml)
		Mean +/- Standard Deviation	Days	Mean +/- Standard Deviation	
IFN	IFN1	811 +/- 154	666	3.5 +/- 3.7	40
	IFN2	767 +/- 215	686	116.6 +/- 170.0	<1.5
	IFN3	977 +/- 254	1000	0.9 +/- 2.5	20
Control	C1	1356 +/- 172	708	3.5 +/- 3.7	<1.5
	C2	777 +/- 189	313	4.3 +/- 3.6	<1.5
	C3	1055 +/- 478	1830	0.8 +/- 0.0	<1.5

### Transduced PBLs

After being transduced with the MFG-K^b^MaIFN-β and MFG-K^b^ΔMaIFN-β constructs, PBLs were readministered to the animal from which they were originally taken. Each macaque was infused with 10^8 ^to 4 × 10^8 ^lymphocytes. Semi-quantitative PCR analysis revealed that the mean transduction efficiencies for the transduction of PBL with the MFG-K^b^MaIFN-β and MFG-K^b^ΔMaIFN-β constructs were 10.33 % ± 7.42 % and 17.13 % ± 10.61 %, respectively. The IFN-β-transduced populations were characterized in culture by a low IFN-β production, ranging from 12 to 24 units per 5 × 10^5 ^cells per 3 days.

We previously published that similar rates of Ma IFN-β-transduction results in a signficant reduction of SIVmac251 replication in vitro [23]. Such IFN-β-transduced cells remain detectable in the blood stream 485 days after reinfusion [24].

After the first inoculation (day 0), transduced cells was detected in peripheral blood, with about 10 transduced cells per 10^6 ^cells, for 14 days in macaque IFN1, and for 29 days in macaques IFN3 and C2 (Table 2). A transient peak of 1000 and 700 transduced cells per 10^6 ^circulating cells was observed in macaques C1 and C3, respectively. After completion of the series of infusions, with the last infusion occurring on day 613, transduced cells persisted at a low level (10 transduced cells per 10^6 ^cells) for only up to 60 days (Table 2). No transduced cells were detected at any time in the study for macaque IFN2. No significant difference was observed between the two groups of macaques in terms of transduced cell persistence (Table 2). The frequency of transduced cells was similar for CD4^+ ^and CD8^+ ^lymphocytes analyzed on day 673 (data not shown). No retroviral construct was detected in lymph nodes and splenic mononuclear cells.

**Table 2:** In vivo follow up of transduced cells in blood. Absolute number of transduced cells per 10^6 ^PBMCs were evaluated by semiquantitative PCR amplification of IFN-β transgene in the two groups of animals. For *in vivo f *ollow up of transduced cells in blood from macaques, DNA samples of PBMCs were obtained at different dates following infusion of transduced PBL. This table indicates the minimum and maximum number of days following the first infusion of transduced cells in which the construct used was still detectable in PBMCs. Moreover, maximum transduction rate of PBMCs and detection treshold of the PCR method are indicated in the two groups of animals. IFN group is represented by the three macaques that received their own cells transduced by the biologically active construct of IFN-β gene whereas the control group is represented by the three macaques that received their own cells transduced by the control one. The relative intensity of the signals was compared to serial dilutions of lysate derived from plasmid-transfected cells that contained known numbers of IFN-β transgene copy per cell. a Day 0 is the first infusion day, other infusions occured at days 361 and 613. b Absolute number of transduced cells was below 10 per 10^6 ^PBMCs.

		1st infusion^a^														
		
Days post-1st infusion		0	1	4	8	12	14	20	22	25	29	33	52	64	81	95
IFN	IFN1	ND^b^	10	10	10	10	10	ND^b^	ND^b^	ND^b^	ND^b^	ND^b^	ND^b^	ND^b^	ND^b^	ND^b^
	IFN2	ND^b^	ND^b^	ND^b^	ND^b^	ND^b^	ND^b^	ND^b^	ND^b^	ND^b^	ND^b^	ND^b^	ND^b^	ND^b^	ND^b^	ND^b^
	IFN3	ND^b^	10	10	10	10	10	10	10	10	10	ND^b^	ND^b^	ND^b^	ND^b^	ND^b^
Control	C1	ND^b^	10	10	1000	100	10	10	10	10	10	ND^b^	ND^b^	ND^b^	ND^b^	ND^b^
	C2	ND^b^	10	10	10	10	10	10	10	10	10	ND^b^	ND^b^	ND^b^	ND^b^	ND^b^
	C3	ND^b^	10	700	10	10	10	10	10	10	10	ND^b^	ND^b^	ND^b^	ND^b^	ND^b^
		2nd infusion^a^								3rd infusion^a^						
				
Days post-1st infusion		361	364	368	375	382	431	489		613	618	625	632	673	688	744
		
IFN	IFN1	ND^b^	10	10	10	ND^b^	ND^b^	ND^b^		ND^b^	ND^b^	ND^b^	ND^b^	ND^b^	ND^b^	ND^b^
	IFN2	ND^b^	ND^b^	ND^b^	ND^b^	ND^b^	ND^b^	ND^b^		ND^b^	ND^b^	ND^b^	ND^b^	ND^b^	ND^b^	ND^b^
	IFN3	ND^b^	10	10	10	ND^b^	ND^b^	ND^b^		ND^b^	10	10	10	10	ND^b^	ND^b^
Control	C1	ND^b^	10	10	10	ND^b^	ND^b^	ND^b^		ND^b^	10	10	10	10	ND^b^	ND^b^
	C2	ND^b^	20	20	20	ND^b^	ND^b^	ND^b^		ND^b^	10	10	10	10	ND^b^	ND^b^
	C3	ND^b^	10	10	10	ND^b^	ND^b^	ND^b^		ND^b^	10	10	10	10	ND^b^	ND^b^

### Clinical status and immunological follow-up

Weight and rectal temperature remained fairly constant throughout the study (data not shown). No major variation in classical hematological parameters, including total lymphocyte and platelets counts, and hemoglobin concentration, was observed (data not shown).

**Figure 1:**
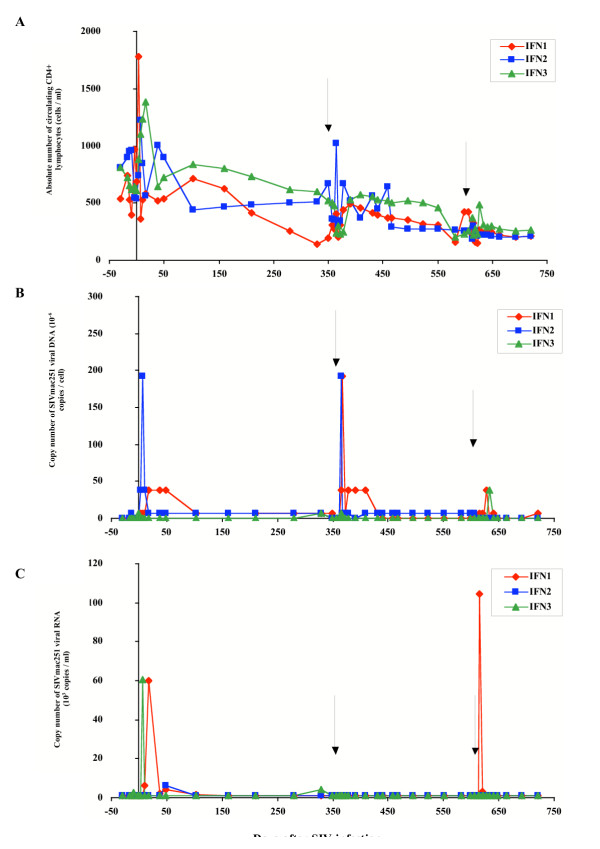
Evolution of immuno-virological parameters in SIVmac251 chronically infected macaques from the IFN group. Immunological and virological parameters were followed in macaques that received their own cells transduced by the retroviral construct allowing expression of the biologically active form of IFN-β. (A) Absolute number of circulating CD4+ lymphocytes was followed by immunophenotyping and flow cytometry. (B) Cell-associated viral load was estimated in PBMCs by a quantitative PCR method based on the specific amplification of the SIV gag gene. (C) Plasma viral load was estimated by a quantitative branched-DNA method based on the specific amplification of the SIV genome. Y axis split X axis at the first reinfusion date (D0) whereas black arrows indicate the second and third reinfusion dates.

**Figure 2:**
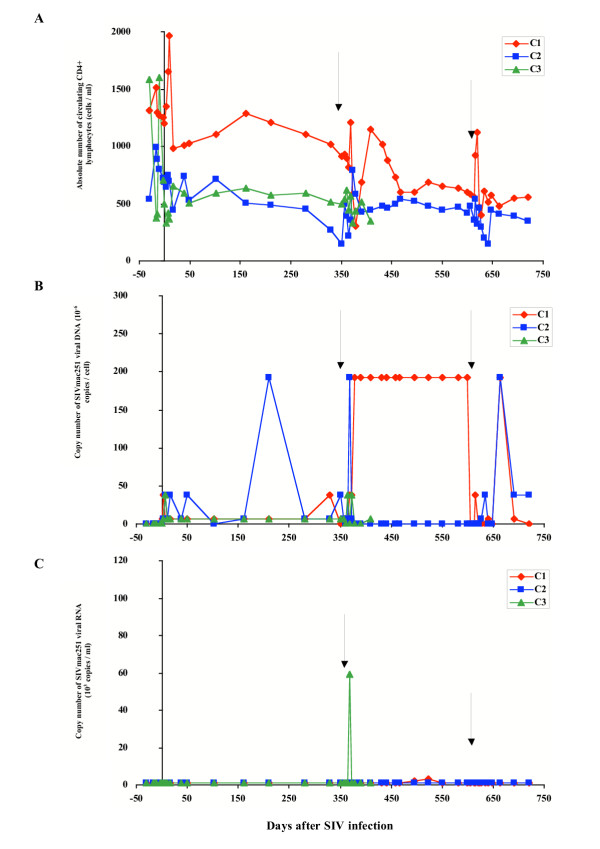
Evolution of immuno-virological parameters in SIVmac251 chronically infected macaques from the control group. Immunological and virological parameters were followed in macaques that received their own cells transduced by the deleted form of the retroviral construct. (A) Absolute number of circulating CD4+ lymphocytes was followed by immunophenotyping and flow cytometry. (B) Cell-associated viral load was estimated in PBMCs by a quantitative PCR method based on the specific amplification of the SIV gag gene. (C) Plasma viral load was estimated by a quantitative branched-DNA method based on the specific amplification of the SIV genome. Y axis split X axis at the first reinfusion date (D0) whereas black arrows indicate the second and third reinfusion dates.

Immunological follow-up indicated that seven days after the first infusion (day 0), the number of circulating CD4^+ ^lymphocytes significantly increased in all macaques studied (p < 0.05), except for C3. A similar significant increase (p < 0.05) was observed in the days following the second infusion (on day 311) for macaques IFN2, and C1, and following the third infusion (on day 613) for macaques IFN1, and C1 (Fig. 1A – 2A).

For all animals in both groups, absolute numbers of CD4+ T cells gradually decreased during the study (p < 0.05), and no significant difference in absolute numbers of CD4+ T cells was observed between the two groups of macaques (Fig. 1A – 2A).

The absolute numbers of circulating CD8^+ ^T lymphocytes increased (p < 0.05) transiently during the days following each infusion of transduced cells, in both groups of macaques. However, with the exception of these peaks, absolute numbers of circulating CD8^+ ^T lymphocytes did not change significantly during the study in any of the animals of either group (data not shown).

### Virological follow-up of animals

We studied the course of SIV infection by determining the number of copies of SIV proviral DNA per cell, and the number of copies of SIV viral RNA per ml of blood. SIV provirus was detected in the PBMCs of all animals in both groups throughout the study. A transient and significant (p < 0.05) increase in cellular viral load was observed one to three weeks after each infusion in macaque IFN1 (Fig. 1B) and in macaques C1, C2 and C3 (Fig. 2B). A similar transient and significant (p < 0.05) increase in cellular viral load also occurred one to three weeks after the first and second infusions in macaque IFN2 and after the third infusion in macaque IFN3 (Fig. 1A).

Analysis of the number of SIV RNA copies in the plasma revealed that plasma viremia peaked (p < 0.05) one week after the first and the third infusions in macaque IFN1 (Fig. 1C), after the first infusion in macaque IFN3 (Fig. 1C) and after the second infusion in macaque C3, (Fig. 2C). The other animals displayed no significant change in plasma viral load during the course of the experiment.

## Discussion

In this study, we assessed the feasibility and efficacy of a gene therapy method based on the introduction into PBL of an IFN-β gene resulting in the constitutive production of low levels of IFN-β, in macaques chronically infected with SIVmac251. The present work was unable to bring new lighting on the efficacy of our gene therapy method since we encountered the problem of disappearence of transduced cells (control or IFN-β transduced cells) few days after each infusion.

Throughout the study, significant, transient peaks of cell-associated and / or plasma viral loads were observed in most animals a few weeks after the infusion of transduced cells. These variations may reflect in vivo activation of viral replication, probably due to the infusion of activated cells. This phenomenon was also observed after the infusion into SCID mice of transduced human PBLs, resulting in up-regulation of CCR-5 HIV co-receptor expression in human CD4^+ ^T cells [27]. Indeed, the SIVmac251 isolate used in our experiment is a CCR5-dependent virus, and its replication may have been activated by upregulation of the CCR-5 coreceptor after infusion. However, gene therapy strategies for the treatment of HIV infection could only be envisaged in combination with HAART. In this context, the activation of host virus replication observed after the infusion of transduced cells would be overcome by HAART treatment.

The mean rates of transduction of PBL isolated from macaques chronically infected with SIVmac251 were 10.33 % ± 7.42 % and 17.13 % ± 10.61 % for the MFG-K^b^MaIFN-β and MFG-K^b^ΔMaIFN-β constructs, respectively, which is similar to the transduction efficiency previously reported for PBLs isolated from healthy non infected macaques [23,24]. The transduction efficacy for lymphocytes from healthy donors and HIV-seropositive patients has also been found to be similar [10], indicating that chronic infection does not affect the retroviral transduction of lymphocytes.

After the first infusion, small numbers of engineered cells (control and IFN-β-transduced cells) were detected for only 29 days. Thus, the persistence of transduced cells in chronically infected macaques was lower than that previously reported in non infected macaques, in which IFN-β-engineered cells were detected for at least 70 days, and for more than a year after SIVmac251challenge [24]. This former study indicates also that immune response that may be induced by mouse cell components or FCS present in culture medium may not alter persistence of genetically modified immune cells. We carried out three infusions of engineered cells and, after each infusion, the engineered cells disappeared from the bloodstream within a few days. Poor persistence of circulating engineered cells has been reported in HIV-infected macaques and in SCID mice, and has been attributed to the delocalization of circulating transduced cells in the lymph nodes [28], and intestine [29]. In our study, we detected no engineered cells in the lymph nodes or spleen, indicating that the delocalization of transduced cells to these organs could not account for the absence of transduced cells in the blood. The short-term persistence of transduced cells has already been reported in other studies in which autologous engineered T cells were cleared rapidly from the bloodstream [30]. However another group reported the persistence of engineered cells for more than 25 weeks (0.1 to 10% of PBMC) in HIV-infected patients [29,31,32]. They hypothesized that the higher rate of T-cell survival was due to *ex vivo *stimulation through CD3 and CD28. Indeed, it has been demonstrated that the inhibition of HIV replication in CD3- CD28- stimulated CD4^+ ^cells is due to the production of cytokines associated with Th-1 function [33] and to the downregulation of CCR-5 expression [34]. Thus, in our study, the disappearance of transduced cells may be due to ConA-stimulation, which may induce apoptosis in lymphocytes, as previously described [35].

IFN-β-producing cells and cells transduced with the control vector displayed similar levels of in vivo persistence. We previously reported higher levels of resistance to HIV in vitro following the transduction of human CD4^+ ^T cells [19], human macrophages [36] and macaque PBL [23] with a construct encoding IFN-β. However, Vieillard et al. [10] reported inefficient protection of transduced lymphocytes against HIV replication in vitro for PBLs isolated from patients in an advanced state of HIV infection. This lack of protection probably resulted from the downregulation of interferon alpha/beta receptor expression in donors with AIDS, leading to hyporesponsiveness to type I IFN [37]. Thus, although we selected animals with CD4^+ ^cell counts that were still high, the disease may have been so advanced that transducing PBLs with a construct encoding IFN-β had little effect, with the engineered lymphocytes subjected to the high rate of lymphocyte turnover observed during SIV infection [38,39].

Our previous work with the macaque model encouraged us to develop low-level autocrine IFN-β production as an approach to gene therapy for AIDS. The persistence of 1 transduced cell per 10^3 ^circulating cells before SIV challenge was correlated with low plasma virus load and the maintenance of CD4^+ ^and CD8^+ ^cell counts in macaques infused with the construct encoding IFN-β [24]. In this study, performed with animals infected for more than one year, cells transduced with the IFN-β construct rapidly disappeared from the bloodstream after infusion. This suggests that gene therapy by PBL transduction should be performed as soon as possible after primary infection. We are well aware that the number of transduced lymphocytes was too small for a major effect in this study and we believe that further exploration of IFN-β-based anti-HIV therapy will require the construction of high-titer vectors, with the aim of increasing the proportion of vector-transduced HIV target cells. An alternative method for IFN-β gene therapy involves the transduction of CD34^+ ^hematopoietic stem cells. This method has been proposed for the treatment of HIV infection [40,41]. The transduction of these cells, which are able to generate all the main HIV target cells, will increase the proportion of transduced cells, extend IFN-β production to macrophages and dendritic cells, and should facilitate long-term expression of the therapeutic construct. We have already demonstrated that macrophages transduced with an IFN-β construct display enhanced HIV resistance, and that HIV transmission to CD4^+ ^T cells is prevented in IFN-β-transduced dendritic cells [42]. We intend to investigate the possibility of transducing hematopoietic stem cells to inhibit viral replication in macaques chronically infected with SIVmac251, in the near future.

## Methods

### Animals

Six male cynomolgus macaques (*Macaca fascicularis*), weighing between 3 and 7 kg, and negative for herpes B, filovirus, STLV-1, SRV-1, SRV-2, SIV, and hepatitis-B were used in this study. Before all experimental procedures, animals were anesthetized with chlorhydrate ketamine (Cenravet, France), and all procedures were conducted according to European guidelines for animal care (Official Journal of the European Communities L538, 18 December 1986). Macaques were housed in individual cages in biosafety level 3 facilities, as required by national regulations (Commission de Génie Génétique, Paris, France).

### Viral stock

More than 300 days before infusion with the IFN construct, macaques were intravenously infected with 4 AID_50 _of a primary, pathogenic SIVmac251 isolate. This virus stock was obtained by coculturing splenocytes obtained from an infected rhesus macaque with rhesus macaque PBMCs (Dr. R.C. Desrosiers, Harvard Medical School, MA, USA), and was amplified by a second passage on rhesus PBMCs (prepared and kindly provided by Dr. A.M. Aubertin, Université Louis Pasteur, Strasbourg France).

### Retroviral vectors

The MFG-K^b^MaIFN-β retroviral vector used in this study has been described elsewhere [23]. It contains the macaque IFN-β coding sequence placed under the control of a 0.6 kb fragment of the murine H2-K^b ^gene promoter, resulting in the continuous production of low levels of a biologically active macaque IFN-β. The MFG-K^b^ΔMaIFN-β retroviral vector used in this study as a control has been described elsewhere [23]. It contains a macaque IFN-β coding sequence with a 530 bp deletion, blocking IFN-β translation, under the control of the same promoter region. Vectors (MFG-K^b^MaIFN-β and MFG-K^b^ΔMaIFN-β were produced with two Ψ-CRIP packaging clones, each of which produced 2 × 10^5 ^infectious particles per ml, with no detectable replication-competent helper virus [23]. The Ψ-CRIP cells were maintained in Dulbecco's modified Eagle's medium (DMEM, InVitrogen, Grand Island, New York, USA) supplemented with 10 % heat-inactivated bovine serum (BS) (InVitrogen) and 0.2 μM antibiotics (penicillin / streptomycin / neomycin, PSN, InVitrogen).

### Isolation of macaque peripheral blood lymphocytes (PBL)

Three macaques (IFN1, IFN2 and IFN3) received infusions of their own lymphocytes transduced with the biologically active MaIFN-β construct. Another three macaques (C1, C2, C3) were infused with their own lymphocytes transduced with the construct carrying the deleted form of the MaIFN-β, which cannot produce a translatable mRNA. We collected about 100 ml of blood from each macaque into heparin lithium tubes (Greiner, USA). Buffy coats were obtained by centrifugation (170 g / 15 min). Mononuclear cells were collected, and centrifuged (400 g / 30 min) on a Ficoll density gradient (Eurobio, Les Ulis, France). Plasma and erythrocytes, diluted 1 in 2 with 0.9% NaCl (InVitrogen), were washed and used immediately for infusion into the macaques.

### Transduction of macaque PBLs

Isolated PBMCs (10^6 ^cells per ml) were activated by incubation for three days in RPMI-1640 medium, 10 % fetal calf serum (FCS), 2 mM L-glutamine (Bœhringer Mannheim, Mannheim, Germany), 0.2 μM antibiotics (penicillin / streptomycin / neomycin), 5 μg / ml concanavalin A (InVitrogen). Activated PBL were resuspended in transduction medium consisting ofn 45 % DMEM, 45 % IMDM (InVitrogen), 5 % FCS, 5 % BS, 4 μg / ml protamine sulfate (Sigma, Saint Louis, USA) and 20 IU / ml recombinant human (rHu) IL-2 (Bœhringer Mannheim). Cells were transduced by coculture for three days with subconfluent Ψ-CRIP packaging cells. At the end of the coculture period, the various cell populations were transferred twice to other culture plates to eliminate any residual adherent packaging cells. Transduced lymphocytes were washed, resuspended in 1× PBS at a concentration of 10^7 ^cells / ml, and injected intravenously into macaques. Transduction efficacy was estimated with transduced PBLs maintained in culture for 3 days.

### Evaluation of the transduction rate

DNA was extracted from macaque PBMCs and the amount used for each sample was normalized based on data for amplification of the β-globin gene, using 5'-ACCATGGTGCTGTCTCCTGC-3' as sense primer, and 5'-CATGGCCACGAGGCTCCA-3' as an antisense primer. Both retroviral sequences were detected, using 5'-GTTCAGGCAAAGTCTTAGTC-3' as the sense primer, binding in the H2-K^b ^gene promoter and 5'-TGAAGATCTCCTAGCCTGT-3 as the antisense primer, binding in the macaque IFN-β coding sequence. These primers amplified a 870-bp fragment from the MFG-K^b^MaIFN-β vector, and a 340-bp fragment from the MFG-K^b^ΔMaIFN- vector The PCR amplification products were identified by dot-blot hybridization with an IFN-β probe, and quantified with a PhosphorImager (Molecular Dynamics, Sevenoaks, England, UK), as previously described [19]. Relative signal intensity was compared with the signal intensity of serial dilutions of lysate derived from plasmid-transfected cells containing known numbers of transgene copies per cell. The detection threshold of the PCR assay used was estimated and found to be one copy of the IFN-β gene per 10^5 ^cells.

### Hematological and immunological follow-up of infused macaques

All infused animals were followed during the months preceding the study, and for more than 700 days after the first autologous infusion. We carried out hematological analysis, and monitored weight, rectal temperature, and levels of lymphocytes transduced with the IFN-β construct. Blood formula and blood cell counts were determined with an automated hemocytometer (Coulter Corporation, Miami, USA). Axillary lymph nodes and spleens were removed from animals and ground in 1× PBS using a Potter homogenizer. Lymph nodes and splenic mononuclear cells (LNMC, SMC) were then collected and centrifuged (400 g / 30 min) on a Ficoll cushion (Eurobio, Les Ulis, France). DNA extraction and evaluation of in the rate of transduction of LNMC and SMC were performed as previously described.

### In vivo immunological follow-up of macaques receiving infusions

We estimated the proportions of the various subtypes of circulating PBMCs by direct immunofluorescence assay (anti-CD3 clone FN18, Biosource International, CA, USA), anti-CD4 clone Leu 3a PE (Becton Dickinson, San Jose, Mountain View, CA, USA), anti-CD8 clone Leu 2a FITC (Becton Dickinson) antibodies and IgG isotypic controls (Immunotech, Marseille, France), and flow cytometry (Becton Dickinson). We used specific software (CellQuest, Becton Dickinson) as previously described [25] for the analysis.

### Sorting of CD4^+ ^and CD8^+ ^circulating lymphocytes

Mononuclear cells isolated on Ficoll-Hypaque were positively separated using CD4-specific and CD8-specific immunomagnetic microbeads (MiniMACS, Miltenyi, Stadt, Germany) according to manufacturer's instructions. Subset purity was evaluated by flow cytometry, using secondary anti-CD4 clone OKT4-PE (Dako, Glostrup, Denmark) and anti-CD8 clone DK25-FITC (Dako) antibodies. The rates of transduction of the sorted CD4^+ ^and CD8^+ ^lymphocytes were evaluated, as described above.

### Plasma and cell-associated viral load

Levels of SIV RNA in plasma were determined with the SIVmac-branched-DNA assay, using a detection threshold of 1,500 mEq per milliliter of plasma (Chiron Diagnostics, Amsterdam, The Netherlands). DNA was extracted from PBMCs with an extraction kit (Roche Diagnostics GmbH, Mannheim, Germany). Levels of SIV DNA in cells were determined using a two-step PCR method with two external *gag*-specific primers (1386-5': GAAACTATGCCAAAAACAAGT and 2129-5': TAATCTAGCCTTCTGTCCTGG) and two internal *gag*-specific primers (1731N 5': CCGTCAGGATCAGATATTGCAGGAA and 2042C 5': CACTAGCTTGCAATCTGGGTT), as previously described [26].

### Statistical analysis

Statistical significance was determined by paired or unpaired non parametric Wilcoxon and Mann-Whitney tests adapted for small samples.

## Competing interests

The authors never received reimbursements, fees, funding, or salary from an organization that may in any way gain or lose financially from the publication of this paper in the past five years. The authors never any stocks or shares in an organization that may in any way gain or lose financially from the publication of this paper. The authors never have any other financial competing interests. The authors have no non-financial competing interests to declare in relation to this paper.

## Authors' contributions

WG was the major contributor to this paper. EL participated in the design of the study and performed the cell cultures and transduction experiments. BB and JL participated in the animals manipulation. FM participated in the preliminary experiments. SP performed all PCR reaction for transduced cells *in vivo *follow-up. DD and EDM participated in the design and the coordination of the study. RLG performed the statistical analysis and participated in the design and the coordination of the study.
